# Circulating cytokine profile and modulation of regulatory T cells in chronic hepatitis B patients with type 2 diabetes mellitus

**DOI:** 10.17305/bjbms.2022.7525

**Published:** 2023-01-06

**Authors:** Wang He, Shu Hao, Xiaohui Dong, Dandan Zhang, Zhen Jia

**Affiliations:** 1 Department of Endocrinology, Xi’an No. 1 Hospital, Xi’an, Shaanxi Province, China

**Keywords:** Chronic hepatitis B, diabetes mellitus, cytokine, interleukin-15, regulatory T cells

## Abstract

The risk of hepatitis B virus (HBV) infection is higher in patients with diabetes mellitus, and diabetes mellitus is one of the metabolic complications of HBV infection. However, the cytokine profile of chronic hepatitis B (CHB) patients with type 2 diabetes mellitus (T2DM) is not fully understood. The aim of this study was to investigate the cytokine expression profile in CHB patients with T2DM, and to assess the regulatory function of cytokines to regulatory T cells (Tregs). Forty-four T2DM patients, 39 CHB patients, 17 patients with CHB and T2DM, and 21 control subjects were enrolled. Cytokine levels in the plasma were measured by Luminex multiplex assay. CD4^+^CD25^+^CD127^dim/−^ Tregs were detected by flow cytometry. Tregs were purified and stimulated with recombinant human interleukin-15 (IL-15). The regulation of IL-15 on Tregs function was investigated by measuring cell number, IL-10/IL-35 secretion, and mRNA expression of immune checkpoint molecules in a Tregs + PBMC co-culture system. We found that levels of IL-1α, IL-6, and IL-33 were upregulated, while interferon-α, IL-2, IL-7, and IL-15 were downregulated in T2DM and CHB patients. CHB patients with T2DM had even lower plasma IL-7 and IL-15 levels. Tregs proportion was elevated in T2DM and CHB patients. CHB patients with T2DM had increased levels of Tregs, which correlated negatively with IL-15. Tregs showed stronger inhibitory activity in CHB patients with T2DM than in controls, T2DM, and CHB patients, which presented as reduction in cellular proliferation and induction of IL-10/IL-35 secretion. IL-15 suppressed Tregs function and inhibited the expression of immune checkpoint molecules in Tregs. The current data suggest that insufficient IL-15 levels and decreased responsiveness of Tregs to IL-15 signaling might contribute to strong immune dysfunction in CHB patients with T2DM.

## Introduction

Hepatitis B virus (HBV) infection is still a global health problem. Worldwide, 257 million people are positive for the hepatitis B surface antigen (HBsAg) [1]. In 2014, the prevalence rate for HBsAg in Chinese population aged 15-29 years was 4.38% [2], indicating approximately 700,000 chronic HBV infections and 200,000-300,000 chronic hepatitis B (CHB) patients [3]. In China, the prevalence of diabetes has significantly increased in recent decades, from 2.5% in the 1990s to 11.6% in 2013, with a high proportion of cases undetected [4]. There is a strong association between liver disease and diabetes, which is higher than expected by a chance association of two common disorders [5]. Three different categories of liver disease could be classified as: liver disease related to diabetes, hepatogenous diabetes, and liver disease occurring coincidentally with diabetes [5]. Individuals with chronic HBV infection have an elevated risk of diabetes [6]. This is partly due to pivotal role of the liver in glucose metabolism [7, 8]. The presence of hepatic disease leads to dysregulation of glucose homeostasis [7]. Meanwhile, the risk of HBV infection is higher in patients who are diagnosed with diabetes mellitus [9, 10]. However, evidence is limited on the effects of diabetes on chronic HBV infection.

The clinical outcome of HBV infection is based on the complicated interaction between the virus and the host immune system [11]. Moreover, both innate and adaptive immunity contribute to the pathogenesis of type 1 diabetes mellitus (T1DM) and type 2 diabetes mellitus (T2DM) [12, 13].

Thus, the interaction between HBV infection and diabetes could have a strong impact on the immune status of patients. Cytokines and chemokines play an essential role in initiating, maintaining, and regulating immunological homeostasis and inflammation in both physiological and pathological processes [14, 15]. Lian et al. showed that several cytokines and chemokines, including interleukin-10 (IL-10), CXCL9, CXCL10, and CXCL11, were increasingly expressed and positively correlated with serum alanine aminotransferase (ALT) in CHB patients [16]. Moreover, CD4 ^+^CD25^high^ regulatory T cells (Tregs) proportion and secreting IL-10 and IL-35 were also elevated in CHB patients, although no statistical correlations were found between Tregs and cytokine expressions [16, 17]. The common γ chain (γC) cytokine family, which includes IL-2, IL-4, IL-7, IL-9, IL-15, and IL-21, is named based on the common usage of their shared receptor subunit γc. γC cytokine family of cytokines plays vital role in the development of innate and adaptive immune cells, promoting cell survival or death of immune populations [18]. γC cytokines regulate T cell response in CHB patients [19]. Elevated IL-7 has effect on the specific cellular immune response in CHB patients [20]. IL-15 directly suppresses HBV replication by interferon-β (IFN-β) *in vivo* [21].

**Table 1:** The clinical characteristics of enrolled subjects

	**Control**	**T2DM**	**CHB**	**CHB+T2DM**
Case (n)	21	44	39	17
Gender (male/female)	11/10	23/21	26/13	10/7
Age (years)	36.0 ± 10.5	40.7 ± 12.4	33.2 ± 8.4	37.1 ± 11.8
Fasting plasma glucose (mmol/L)	5.02 ± 0.84	12.87 ± 3.16	4.88 ± 1.02	11.06 ± 2.73
HbA1c (%)	5.05 ± 0.91	7.63 ± 0.62	5.38 ± 1.14	7.04 ± 0.67
ALT (IU/L)	23 (11, 30)	32 (16, 39)	97 (69, 176)	88 (71, 142)
HBV DNA (log10IU/mL)	Not detectable	Not detectable	6.07 ± 1.24	5.88 ± 0.95
HBsAg positive	0	0	39	17
anti-HBs positive	17	33	0	0
HBeAg positive	0	0	27	12
anti-HBe positive	0	0	10	4
HBcAg positive	0	0	39	17

T2DM: Type 2 diabetes mellitus; CHB: Chronic hepatitis B; HbA1c: Glycated haemoglobin; ALT: Alanine aminotransferase; HBsAg: Hepatitis B surface antigen; HBeAg: Hepatitis B e antigen; HBcAg: Hepatitis B core antigen.

Inayat et al. revealed that inflammation-associated genes were upregulated in peripheral blood leukocytes in T2DM patients, while metformin was demonstrated to be an anti-inflammatory reagent, that appeared to be independent of its anti-hyperglycemic activity in T2DM patients [22]. IL-7 levels are increased in gingival crevicular fluid and saliva and in clinical periodontal parameters of middle-aged and elderly T2DM patients [23]. Elevated IL-15 expression alters the biological behavior of trophoblasts *in vitro* and contributes to the placental pathology in gestational diabetes mellitus [24]. Furthermore, gestational diabetes also shows a significantly different peripheral T helper (Th) cell profile, characterized by a higher proportion of Th2, Th17, and Tregs [25].

Few studies focused on cytokine expression and Tregs regulation in CHB patients with T2DM. In the present study, we investigated the circulating cytokine expression profile by Luminex multiplex assay and assessed the modulatory role of differentially expressed cytokines on Tregs in CHB patients with T2DM using an *in vitro* cell culture system.

## Materials and Methods

### Study population and definition

There were four groups involved in the current study: Control group, T2DM group, CHB group, and CHB + T2DM group. Control groups were healthy individuals who had a health examination in our hospital. T2DM patients were defined as having typical symptoms of diabetes with random blood glucose testing ≥11.1 mmol/L, or with fasting plasma glucose ≥7.0 mmol/L, or with oral glucose tolerance testing ≥11.1 mmol/L, or with glycated hemoglobin (HbA1c) ≥6.5%. CHB patients were defined as HBsAg and HBV DNA positive for more than six months, with elevated serum ALT. None of the subjects had malignant tumors, end-stage liver disease, or autoimmune disorders. Individuals who were co-infected with human immunodeficiency virus or other active hepatitis virus infection were excluded from the study. None of the patients received anti-diabetic or anti-viral therapy before sampling. Ten milliliters of anti-coagulant peripheral blood were obtained. The clinical characteristics of enrolled subjects are shown in Table 1.

### Plasma cytokine detection

Plasma was isolated by centrifugation at 1000 *r*/min for 10 min and kept at −80 ^∘^C until use. A total of 14 cytokines were selected as target cytokines, including 4 important γC cytokines (IL-2, IL-4, IL-7, and IL-15), 9 key cytokines related to CHB, and diabetes mellitus cellular immunity (IFN-α, IFN-γ, IL-1α, IL-1β, IL-1 receptor α [IL-1ra], IL-3, IL-6, IL-33, and vascular endothelial growth factor [VEGF]), and the key cytokine related to Tregs function (IL-10). Plasma cytokine levels were measured by Human Cytokine Magnetic Luminex Performance Assay 14-Plex Fixed Panel (R&D systems, Minneapolis, MN, USA; Catalog# LKTM011) using Luminex 200 Multiplexing Instrument (EMD Millipore, Billerica, MA, USA) following manufacturer’s instructions.

### Flow cytometry analysis

Peripheral blood mononuclear cells (PBMCs) were prepared by density gradient centrifugation method using Human Lymphocyte Separation Medium (Solarbio Life Science, Beijing, China; Catalog# P8610), and were kept in liquid nitrogen until use. The freezing medium for PBMCs was 90% fetal bovine serum supplemented with 10% dimethyl sulfoxide in a concentration of 10^8^ cells per vial. PBMCs were stained with allophycocyanin (APC) Mouse Anti-Human CD3 (BD Pharmingen, San Jose, CA, USA; Clone SP34-2; Catalog# 557597), peridinin-chlorophyll-protein complex (PerCP) Mouse Anti-Human CD4 (BD Pharmingen, San Jose, CA, USA; Clone L200; Catalog# 550631), fluorescein isothiocyanate (FITC) Mouse Anti-Human CD25 (BD Pharmingen, San Jose, CA, USA; Clone M-A251; Catalog# 555431), and phycoerythrin (PE) Mouse Anti-Human CD127 (BD Pharmingen, San Jose, CA, USA; Clone HIL-7R-M21; Catalog# 560822) for 30 minutes in the dark. Cells were analyzed by BD FACS Calibur Flow Cytometer (BD Bioscience, San Jose, CA, USA).

### Isolation, purification, stimulation, and culture of Tregs

CD4^+^CD25^+^CD127^dim/−^ Tregs were purified from PBMCs by CD4^+^CD25^+^CD127^dim/−^ Regulatory T Cell Isolation Kit II, human (Miltenyi Biotec, Bergisch Gladbach, Germany; Catalog# 130-094-775) using magnetic activated cell separation method following manufacturer’s instructions. CD4^+^CD25^+^CD127^dim/−^ Tregs were stimulated with recombinant human IL-15 protein (R&D Systems, Minneapolis, MN, USA; Catalog# 247-ILB-025/CF) at the final concentration of either 10 ng/mL or 100 ng/mL for 48 hours. Purified Tregs amount of 5 × 10^4^ was co-cultured in direct contact with 2 × 10^5^ of autologous PBMCs for another 72 hours in the presence of anti-CD3/CD28 (1 µg/mL) as previously reported [26]. The total cell number in the co-culture system was determined with the Cell Counting Kit 8 (WST-8/CCK-8) (Abcam, Cambridge, MA, USA; Catalog# ab228554) following manufacturer’s instructions. A 20 µL of CCK-8 solution was added to each well in the past 4 hours of culturing. Absorbance of the samples was measured at 450 nm. Wells containing a known number of viable PBMCs were used to create a calibration curve for calculation of tested cell numbers.

### IL-10 and IL-35 level detection

Treg-secreting cytokines, including IL-10 and IL-35, were measured in the cultured supernatants by commercial enzyme-linked immunosorbent assay (ELISA) kits (CUSABIO, Wuhan, Hubei Province, China; Catalog# CSB-E04593h and CSB-E13126h) following manufacturer’s instructions.

### mRNA expression detection in Tregs

mRNA expression of IL-15 receptor α chain (IL-15Rα), cytotoxic T-lymphocyte-associated protein 4 (CTLA-4), lymphocyte-activation gene 3 (LAG-3), programmed death-1 (PD-1), and T-cell immunoglobulin and mucin domain-3 (TIM-3) were detected in Tregs. Total RNA was extracted from CD4^+^CD25^+^CD127^dim/−^ Tregs by RNeasy Mini Kit (QIAGEN, Hilden, Germany; Catalog# 74106) following manufacturer’s instructions. A 1 µg of total RNA was reversely transcripted to cDNA by PrimeScript RT reagent Kit (Perfect Real Time) (TaKaRa, Beijing, China; Catalog# RR037A). Real-time polymerase chain reaction was performed by TB Green *Premix Ex Taq* II (Tli RNaseH Plus) (TaKaRa, Beijing, China; Catalog# RR820A) using Applied Biosystems 7500 Real-Time PCR System (Applied Biosystems, Foster, CA, USA). mRNA relative level for each target gene was semi-quantified using 2*^−ΔΔCT^* method. The primer sequences are shown in Table 2.

**Table 2:** Primer sequences for real-time polymerase chain reaction

**Primer**	**Sequence**
IL-15Rα forward	5’-AACAGCCAAGAACTGGGAACT-3’
IL-15Rα reverse	5’-TTGCCTTGACTTGAGGTAGCAT-3’
CTLA-4 forward	5’-GCCCTGCACTCTCCTGTTTTT-3’
CTLA-4 reverse	5’- GGTTGCCGCACAGACTTCA-3’
LAG-3 forward	5’-GCGGGGACTTCTCGCTATG-3’
LAG-3 reverse	5’-GGCTCTGAGAGATCCTGGGG-3’
PD-1 forward	5’-CCAGGATGGTTCTTAGACTCCC-3’
PD-1 reverse	5’-TTTAGCACGAAGCTCTCCGAT-3’
TIM-3 forward	5’-TCCAAGGATGCTTACCACCAG-3’
TIM-3 reverse	5’-GCCAATGTGGATATTTGTGTTAGATT-3’
β-actin forward	5’-GGCACCCAGCACAATGAAG-3’
β-actin reverse	5’-CGTCATACTCCTGCTTGCTG-3’

IL-15Rα: IL-15 receptor α chain; CTLA-4: Cytotoxic T-lymphocyte-associated protein 4; LAG-3: Lymphocyte-activation gene 3; PD-1: Programmed death-1; TIM-3: T-cell immunoglobulin and mucin domain-3.

### Ethical statement

The protocol was approved by the Ethics Committee of Xi’an No.1 Hospital. The study was performed in agreement with the Declaration of Helsinki. Each participant signed an informed consent form.

### Statistical analysis

All data were analyzed using SPSS version 23.0 for Windows (Chicago, IL, USA). Shapiro–Wilk test was used for normal distribution assay. Variables following normal distribution were presented as mean ± standard deviation (SD). Statistical significance was determined by one-way analysis of variance (ANOVA) and Student-Newman-Keuls (SNK)-*q* test. Variables following skewed distribution were presented as median with interquartile range (M [Q1, Q3]). Statistical significance was determined by Kruskal–Wallis *H* test and Dunn’s multiple comparison test. Pearson correlation analysis was performed for correlation analysis. A value of *p* < 0.05 was considered to indicate a significant difference.

## Results

### Circulating cytokine expression profile

As shown in Table 3, all 14 cytokines could be detected in the plasma from all enrolled subjects. There were no significant differences in IFN-γ, IL-1β, IL-1ra, IL-3, IL-4, IL-10, or VEGF in the plasma between four groups (*p* > 0.05, Table 3). The CHB group and the CHB + T2DM group had elevated peripheral IL-1α levels in comparison to the control group (*p* < 0.05, Table 3), whereas IFN-α levels were reduced compared with the control group (*p* < 0.05, Table 3). Plasma IFN-α was also decreased in the CHB group and the CHB + T2DM group when compared to the T2DM group (*p* < 0.05, Table 3). IL-6 and IL-33 levels were significantly upregulated, while IL-2, IL-7, and IL-15 levels were significantly downregulated in the T2DM group, CHB group, and CHB + T2DM group compared with control group (*p* ═ 0.001, Table 3). Importantly, IL-7 and IL-15 levels in the CHB + T2DM group were notably downregulated compared with the T2DM and CHB groups (*p* < 0.05, Table 3). Thus, we analyzed the function of IL-7 and IL-15 in CHB + T2DM patients in further experiments.

**Table 3:** Cytokine levels in the plasma from enrolled subjects (pg/mL)

	**Control (*n* ═ 21)**	**T2DM (*n* ═ 44)**	**CHB (*n* ═ 39)**	**CHB+T2DM (*n* ═ 17)**	**Statistics value**	***p*-value**
IFN-α	212.4 ± 78.30	198.3 ± 67.02	161.8 ± 58.72*^#^	162.1 ± 49.41*^#^	*F* ═ 4.279	0.007
IFN-γ	17.43 (8.82, 40.67)	15.44 (8.26, 32.83)	13.20 (6.86, 20.75)	13.67 (6.23, 38.78)	*H* ═ 3.032	0.183
IL-1α	11.02 (4.42, 21.78)	15.63 (9.88, 29.73)	17.10 (11.04, 26.080)*	17.10(9.32, 26.91)*	*H* ═ 7.572	0.024
IL-1β	5.88 (3.67, 8.91)	6.04 (4.64, 8.95)	5.68 (3.31, 7.36)	5.92 (4.09, 9.24)	*H* ═ 4.274	0.109
IL-1ra	27.88 ± 6.34	24.68 ± 7.71	26.41 ± 6.67	29.31 ± 8.36	*F* ═ 2.022	0.115
IL-2	287.4 ± 57.02	228.0 ± 67.31*	237.3 ± 68.28*	202.4 ± 72.11*	*F* ═ 5.699	0.001
IL-3	131.2 ± 38.41	137.0 ± 30.49	141.8 ± 44.26	139.4 ± 29.74	*F* ═ 0.397	0.755
IL-4	45.29 ± 10.46	48.92 ± 11.97	52.28 ± 9.27	51.33 ± 9.04	*F* ═ 2.233	0.088
IL-6	56.30 ± 18.04	67.81 ± 22.70*	70.89 ± 20.31*	73.93 ± 21.94*	*F* ═ 2.837	0.041
IL-7	78.21 ± 10.08	63.47 ± 15.23*	60.78 ± 14.72*	48.65 ± 13.61*^#$^	*F* ═ 14.39	<0.001
IL-10	436.2 (302.1, 576.4)	472.4 (296.1, 602.8)	474.0 (337.8, 552.9)	459.8 (349.2, 632.8)	*H* ═ 1.676	0.183
IL-15	306.7 ± 78.02	263.7 ± 57.31*	267.9 ± 72.39*	224.2 ± 51.77*^#$^	*F* ═ 5.005	0.003
IL-33	1782 ± 492.3	2083 ± 502.7*	2297 ± 673.4*	2301 ± 583.1*	*F* ═ 4.293	0.007
VEGF	22.08 (10.31, 31.31)	18.34 (9.02, 38.19)	17.22 (8.60, 27.70)	19.27 (13.10, 30.83)	*H* ═ 1.786	0.412

F refers to the statistical value for one-way ANOVA, while H refers to the statistical value for Kruskal–Wallis H test. *p < 0.05 compared with control; ^#^*p* < 0.05 compared with T2DM; ^$^*p* < 0.05 compared with CHB. T2DM: Type 2 diabetes mellitus; CHB: Chronic hepatitis B; IFN: Interferon; IL: Interleukin; VEGF: Vascular endothelial growth factor.

### Tregs detection before and after PBMCs co-culture

**Figure 1.**
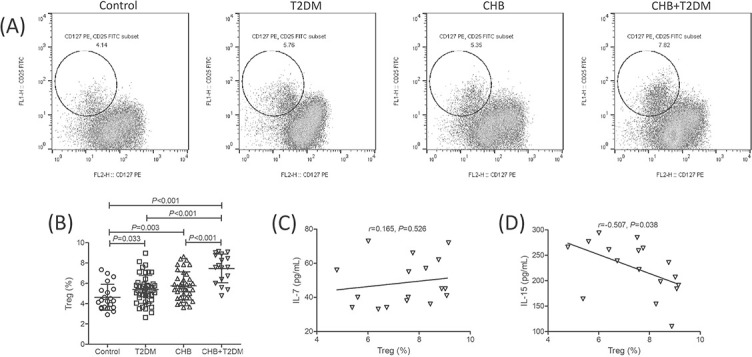
**CD4^+^CD25^+^CD127^dim/−^ Tregs analysis in control (*n* ═ 21), T2DM (*n* ═ 44), CHB (*n* ═ 39), and CHB+T2DM group (*n* ═ 17).** (A) CD4^+^CD25^+^CD127^dim/−^ Tregs were analyzed by flow cytometry. PBMCs were stained with anti-CD3-APC, anti-CD4-PerCP, anti-CD25-FITC, and anti-CD127-PE. The flow dots for CD25^+^CD127^dim/−^ cells within CD3^+^CD4^+^ cells in the control, T2DM, CHB, and CHB+T2DM group were shown. (B) CD4^+^CD25^+^CD127^dim/−^ Tregs proportion within CD3^+^CD4^+^ cells was compared between the control, T2DM, CHB, and CHB+T2DM groups. Statistical analysis was performed using one-way ANOVA and SNK-*q* test. (C) Correlation between Tregs proportion and plasma IL-7 levels was analyzed in the CHB+T2DM group. (D) Correlation between Tregs proportion and plasma IL-15 levels was analyzed in the CHB+T2DM group. Pearson correlation analysis was performed for correlation analysis. T2DM: Type 2 diabetes mellitus; CHB: Chronic hepatitis B; Tregs: Regulatory T cells; IL: Interleukin; PBMCs: Peripheral blood mononuclear cells.

CD4^+^CD25^+^CD127^dim/−^ Tregs were analyzed by flow cytometry, and representative flow dots for Tregs analysis in each group are shown in Figure 1A. CD4^+^CD25^+^CD127^dim/−^ Tregs proportion within CD3^+^CD4^+^ T cells was significantly elevated in both T2DM group (5.41 ± 1.37%) and CHB group (5.75 ± 1.38%) compared with the control group (4.63 ± 1.27%) (*p* < 0.05, Figure 1B). Importantly, Tregs proportion was significantly increased in the CHB + T2DM group (7.47 ± 1.42%) compared with that in the control, T2DM, and CHB group (*p* < 0.001, Figure 1B). In the view of the significant downregulation of plasma IL-7 and IL-15, we further observed the correlation between IL-7/IL-15 level and Tregs in the CHB + T2DM group. There was no remarkable correlation between Tregs proportion and plasma IL-7 level in the CHB + T2DM group (*r* ═ 0.165, *p* ═ 0.526, Figure 1C). However, Tregs proportion was negatively correlated with plasma IL-15 level in the CHB + T2DM group (*r* ═ −0.507, *p* ═ 0.038, Figure 1D).

**Figure 2.**
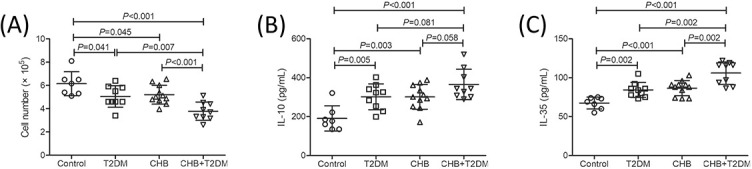
**Inhibitory activity analysis of CD4^+^CD25^+^CD127^dim/−^ Tregs in the control (*n* ═ 7), T2DM (*n* ═ 9), CHB (*n* ═ 11), and CHB+T2DM group (*n* ═ 9).** 5 × 10^4^ of purified CD4^+^CD25^+^CD127^dim/−^ Tregs were co-cultured with 2 × 10^5^ of autologous PBMCs for 72 hours. (A) Cell number was determined by CCK-8 method and was compared between the control, T2DM, CHB, and CHB+T2DM groups. (B) IL-10 levels in the cultured supernatants were measured by ELISA and were compared between the control, T2DM, CHB, and CHB+T2DM groups. (C) IL-35 levels in the cultured supernatants were measured by ELISA and were compared between the control, T2DM, CHB, and CHB+T2DM groups. Statistical analysis was performed using one-way ANOVA and SNK-*q* test. T2DM: Type 2 diabetes mellitus; CHB: Chronic hepatitis B; Tregs: Regulatory T cells; IL: Interleukin; PBMCs: Peripheral blood mononuclear cells.

CD4^+^CD25^+^CD127^dim/−^ Tregs were purified from 7 controls, 9 T2DM patients, 11 CHB patients, and 9 CHB + T2DM patients. Purified Tregs amount of 5 × 10^4^ was co-cultured with 2 × 10^5^ of autologous PBMCs for 72 hours. Total cell number from controls was significantly higher than in T2DM patients, CHB patients, and CHB + T2DM patients (*p* < 0.05, Figure 2A). Importantly, cell number in CHB + T2DM patients was significantly lower than in T2DM (*p* ═ 0.007, Figure 2A) and CHB patients (*p* < 0.001, Figure 2A). This result suggested that the inhibitory function of Tregs was the most significant in the T2DM + CHB group, which was consistent with the highest proportion of Tregs in this group before culturing.

**Figure 3.**
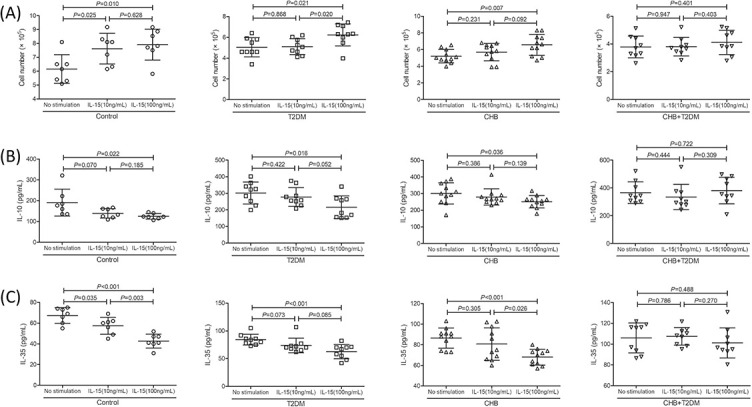
**Inhibitory activity analysis of CD4^+^CD25^+^CD127^dim/−^ Tregs in response to IL-15 stimulation in the control (*n* ═ 7), T2DM (*n* ═ 9), CHB (*n* ═ 11), and CHB+T2DM group (*n* ═ 9).** Purified CD4^+^CD25^+^CD127^dim/−^ Tregs were stimulated with either 10 ng/mL or 100 ng/mL recombinant human IL-15 for 24 hours. 5×  10^4^ of stimulated Tregs were co-cultured with 2 × 10^5^ of autologous PBMCs for 72 hours. (A) Cell number was determined by CCK-8 method and was compared between no stimulation, 10 ng/mL of IL-15 stimulation, and 100 ng/mL of IL-15 stimulation in each group. (B) IL-10 levels in the cultured supernatants were measured by ELISA and were compared between no stimulation, 10 ng/mL of IL-15 stimulation, and 100 ng/mL of IL-15 stimulation in each group. (C) IL-35 levels in the cultured supernatants were measured by ELISA and were compared between no stimulation, 10 ng/mL of IL-15 stimulation, and 100 ng/mL of IL-15 stimulation in each group. Statistical analysis was performed using one-way ANOVA and SNK-*q* test. T2DM: Type 2 diabetes mellitus; CHB: Chronic hepatitis B; Tregs: Regulatory T cells; IL: Interleukin.

Due to the potential correlation of IL-15 levels and Tregs proportion in the CHB + T2DM group, CD4^+^CD25^+^CD127^dim/−^ Tregs were stimulated with either 10 ng/mL or 100 ng/mL recombinant human IL-15 for 24 hours to analyze the response of IL-15 in different groups. Stimulated Tregs were co-cultured with autologous PBMCs for another 72 hours. Both 10 ng/mL and 100 ng/mL of IL-15 stimulation strongly increased cell number in controls (*p* < 0.05, Figure 3A). Only 100 ng/mL of IL-15 promoted cellular proliferation in T2DM (*p* ═ 0.021, Figure 3A) and CHB patients (*p* ═ 0.007, Figure 3A), while 10 ng/mL of IL-15 did not affect cell number in either group (*p* > 0.05, Figure 3A). However, cell number did not change significantly in CHB + T2DM patients with either 10 ng/mL or 100 ng/mL of IL-15 stimulation (*p* > 0.05, Figure 3A).

### IL-10 and IL-35 levels in supernatants before and after IL-15 stimulation

Purified Tregs amount of 5 × 10^4^ was co-cultured with 2 × 10^5^ of autologous PBMCs for 72 hours. Both IL-10 and IL-35 levels in the cultured supernatants were significantly lower in control compared with those in T2DM patients, CHB patients, and CHB + T2DM patients (*p* < 0.05, Figure 2B and 2C). Although IL-10 level was slightly elevated in CHB + T2DM patients (365.4 ± 77.79 pg/mL) compared with T2DM (301.9 ± 66.38 pg/mL) and CHB patients (301.4 ± 63.78 pg/mL), these differences failed to achieve statistical significance (*p* ═ 0.081 and 0.058, respectively, Figure 2B). IL-35 level was notably higher in CHB + T2DM patients (106.0 ± 14.40 pg/mL) than in T2DM (84.31 ± 9.80 pg/mL) and CHB patients (86.55 ± 9.77 pg/mL) (*p* ═ 0.002, Figure 2C).

Similarly, although IL-10 downregulation in response to 10 ng/mL of IL-15 stimulation failed to achieve significant difference in controls (*p* ═ 0.070, Figure 3B), IL-10 and IL-35 secretion in response to IL-15 stimulation seemed strongly suppressed in controls (*p* < 0.05, Figure 3B and 3C). Only 100 ng/mL of IL-15 inhibited IL-10 and IL-35 expression in T2DM (*p* < 0.05, Figure 3B and 3C) and CHB patients (*p* < 0.05, Figure 3B and 3C), while 10 ng/mL of IL-15 did not affect IL-10 and IL-35 production in either group (*p* > 0.05, Figure 3B and 3C). Neither IL-10 nor IL-35 expression notably changed in CHB + T2DM patients with 10 ng/mL and 100 ng/mL of IL-15 stimulation (*p* > 0.05, Figure 3B and 3C).

### Immune checkpoint molecules expression in Tregs after IL-15 stimulation

**Figure 4.**
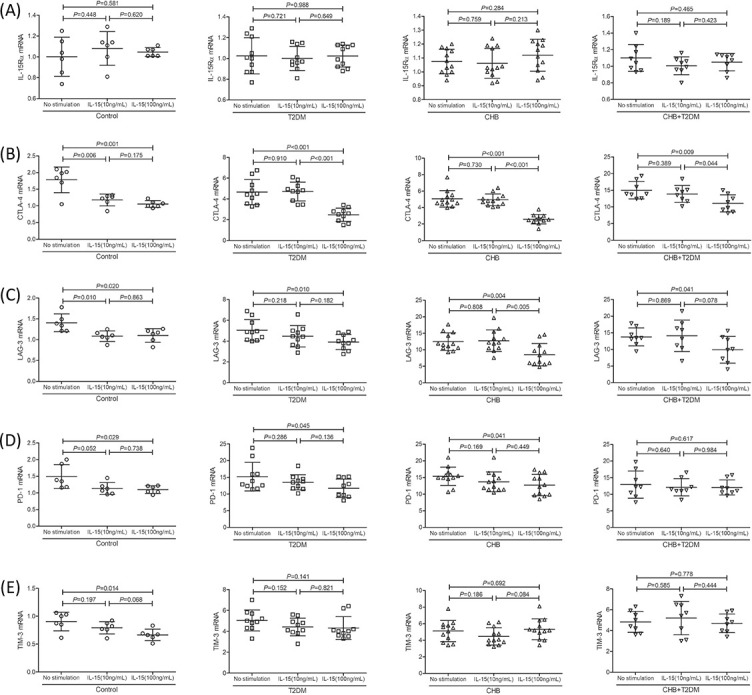
**The influence of IL-15 stimulation to IL-15Rα and immune checkpoint molecules expression in CD4^+^CD25^+^CD127^dim/−^ Tregs in the control (*n* ═ 6), T2DM (*n* ═ 10), CHB (*n* ═ 12), and CHB+T2DM group (*n* ═ 8).** Purified CD4^+^CD25^+^CD127^dim/−^ Tregs were stimulated with either 10 ng/mL or 100 ng/mL recombinant human IL-15 for 24 hours. mRNA relative levels corresponding to IL-15Rα, CTLA-4, LAG-3, PD-1, and TIM-3 were semi-quantified by real-time PCR. (A) IL-15Rα mRNA relative levels were compared between no stimulation, 10 ng/mL of IL-15 stimulation, and 100 ng/mL of IL-15 stimulation in each group. (B) CTLA-4 mRNA relative levels were compared between no stimulation, 10 ng/mL of IL-15 stimulation, and 100 ng/mL of IL-15 stimulation in each group. (C) LAG-3 mRNA relative levels were compared between no stimulation, 10 ng/mL of IL-15 stimulation, and 100 ng/mL of IL-15 stimulation in each group. (D) PD-1 mRNA relative levels were compared between no stimulation, 10 ng/mL of IL-15 stimulation, and 100 ng/mL of IL-15 stimulation in each group. (E) TIM-3 mRNA relative levels were compared between no stimulation, 10 ng/mL of IL-15 stimulation, and 100 ng/mL of IL-15 stimulation in each group. Statistical analysis was performed using one-way ANOVA and SNK-*q* test. T2DM: Type 2 diabetes mellitus; CHB: Chronic hepatitis B; Tregs: Regulatory T cells; IL: Interleukin.

CD4^+^CD25^+^CD127^dim/−^ Tregs were purified from 6 controls, 10 T2DM patients, 12 CHB patients, and 8 CHB + T2DM patients and were stimulated with either 10 ng/mL or 100 ng/mL recombinant human IL-15 for 24 hours to analyze the potential mechanism of IL-15-induced Tregs regulation. mRNA expressions corresponding to IL-15Rα and immune checkpoint molecules were semi-quantified by real-time PCR. There was no remarkable difference in IL-15Rα mRNA relative levels in Tregs between four groups (*p* > 0.05). L-15R mRNA relative levels in Tregs exposed to no stimulation, 10 ng/mL of IL-15 stimulation, or 100 ng/mL of IL-15 stimulation did not differ significantly between groups (*p* > 0.05, Figure 4A). mRNA expressions of immune checkpoint molecules, including CTLA-4, LAG-3, PD-1, and TIM-3, were significantly increased in the T2DM, CHB, and CHB + T2DM group compared with control group (*p* < 0.05). Both 10 ng/mL and 100 ng/mL of IL-15 stimulation reduced CTLA-4 and LAG-3 mRNA expression in controls (*p* < 0.05, Figure 4B and 4C). However, only 100 ng/mL of IL-15 induced downregulation of CTLA-4 and LAG-3 mRNA in T2DM, CHB, and CHB + T2DM patients (*p* < 0.05, Figure 4B and 4C). 10 ng/mL of IL-15 did not affect PD-1 mRNA relative levels in Tregs in any group (*p* > 0.05, Figure 4D), while 100 ng/mL of IL-15 dampened PD-1 mRNA expression in controls, T2DM, and CHB patients (*p* < 0.05, Figure 4D). However, neither 10 ng/mL nor 100 ng/mL induced PD-1 downregulation in Tregs in CHB patients with T2DM (*p* > 0.05, Figure 4D). 100 ng/mL of IL-15 slightly reduced TIM-3 mRNA expression in controls (*p* ═ 0.014, Figure 4E). However, IL-15 did not affect TIM-3 mRNA relative level in Tregs in T2DM, CHB, and CHB + T2DM patients (*p* > 0.05, Figure 4E).

## Discussion

Herein, the current results indicate a different peripheral cytokine expression profile in CHB patients with T2DM compared with CHB patients and T2DM patients. IL-6 and IL-33 levels were increased in both CHB and T2DM patients; however, levels of both cytokines were not elevated in CHB patients with T2DM. Importantly, three members of the γC cytokine family, including IL-2, IL-7, and IL-15, were extensively studied. Plasma IL-2, IL-7, and IL-15 levels were downregulated in both CHB and T2DM patients. IL-7 and IL-15 expression was further suppressed in CHB patients with T2DM. Although reduced IL-2 expression in CHB patients with T2DM did not achieve statistical difference compared with CHB and T2DM patients (*p* ═ 0.082 and *p* ═ 0.071), the levels were still slightly decreased. This might be partly due to the limited enrollment of patients, and the results still need to be confirmed in larger sample size. Taken together, insufficient γC cytokines secretion might contribute to the pathogenesis of chronic HBV infection with T2DM.

IL-7 is a potent proliferation, activation, and survival cytokine for T cell that enhances antiviral and antitumor responses through its receptor IL-7 α chain (CD127) [27]. Circulating IL-7 levels were decreased in patients with chronic hepatitis C (HCV) infection and were negatively correlated with viral replication and liver inflammation [28, 29]. Exogenous IL-7 enhanced HCV-specific and non-specific CD8^+^ T cell and T follicular helper (Tfh) cell function, which contributed to the viral clearance [28, 29]. IL-7 levels in CHB patients are also closely related to Tfh cell activity. IL-7 could elevate Tfh and HBV-specific cellular immune responses, thereby reducing HBV DNA *in vitro* [20]. Similarly, diabetes was associated with decreased tyrosine nitrosylation of IL-7, which was associated with diabetic retinopathy [30]. However, HbA1c was found to be positively correlated with IL-7 in overweight/obese young subjects with prediabetes [31]. Importantly, Mohamed et al. demonstrated that T2DM patients with chronic periodontitis had lower IL-7 levels in gingival crevicular fluid compared with both T2DM and chronic periodontitis patients [32]. We found similar IL-7 expression profile in CHB patients with T2DM, which was further reduced in comparison to both T2DM and CHB patients, suggesting a synergistic effect of T2DM and chronic HBV infection on suppression of IL-7 expression.

IL-15 plays an important role in development, survival, and activation of natural killer (NK) cells, and in maintenance of memory CD8^+^ T cell homeostasis through IL-15Rα signaling pathway [33]. Anti-HBV therapy induced upregulation of IL-15 in CHB patients, which functionally restored CD56^bright^ NK cells [34]. IL-15 also contributed to *in vivo* HBV clearance through multiple mechanisms, including the complementary effects on IFN-α [35], induction of IFN-β production [21], and enhancement of CD8^+^ T cells longevity [36]. However, IL-15 fluctuation in the liver during chronic woodchuck hepatitis virus infection was not associated with viral load [37]. IL-15 was decreased in T2DM patients [38]. IL-15 administration inhibited the negative effects of tumor necrosis factor-α in T2DM patients [39], indicating the beneficial metabolic activities of IL-15 in T2DM [40]. Importantly, Sánchez-Jiménez et al. showed strong association between IL-15 and insulin levels in patients with pulmonary tuberculosis; however, this association was weaker in pulmonary tuberculosis patients with T2DM [41]. Our present data suggest the similar expression profile of IL-15 and IL-7. The extreme decrease of IL-15 in CHB patients with T2DM might be due to the synergistic effects of chronic HBV infection and diabetes. Interestingly, IL-15 level was negatively correlated with CD4^+^CD25^+^CD127^dim/−^ Tregs proportion in CHB patients with T2DM, indicating a possible regulatory activity of IL-15 on Tregs in CHB patients with T2DM.

CD4^+^CD25^+^CD127^dim/−^ Tregs exhibited immunosuppressive activity in CHB patients, which was important for persistent HBV infection [42]. However, controversy remained as to the proportion and function of Tregs in T2DM. The proportion of Tregs seemed to be downregulated in T2DM [43], which was associated with the disease progression [44] and decreased regulatory function in response to the IL-2 signaling pathway [45]. Zhang et al. reported that although Tregs proportion did not change significantly, there was a negative correlation between Tregs peripheral proportion and urine albumin/creatinine ratio in T2DM patients [46]. We found that CD4^+^CD25^+^CD127^dim/−^ Tregs proportion was significantly increased in both T2DM and CHB patients, while Tregs proportion was further significantly elevated in CHB patients with T2DM, suggesting the synergistic effect of persistent HBV infection and diabetes on immunosuppressive activity of Tregs. Importantly, Tregs proportion was negatively associated with IL-15, but not with IL-7. Due to the low or negative expression of CD127 in Tregs, it was also assumed that IL-7 signaling might not contribute to Tregs function.

The regulation of IL-15 to Tregs in CHB patients with T2DM was then assessed. Two different concentrations of IL-15, 10 ng/mL, and 100 ng/mL were used. Interestingly, both concentrations of IL-15 suppressed Tregs function in controls, while only higher concentration of IL-15 inhibited Tregs activity in CHB and T2DM patients. Unfortunately, neither concentration of IL-15 could regulate Tregs function in CHB patients with T2DM. This indicated a decreased responsiveness of Tregs to IL-15 in these patients. We thus investigated the potential mechanisms for this reduced responsiveness to IL-15. CD4^+^CD25^+^CD127^dim/−^ Tregs exerted suppressive function through various mechanisms, including the requirement of cell-to-cell contact, inhibitory cytokine secretion, and potential cytotoxic activity [47]. First, there was no significant difference of IL-15Rα expression in Tregs between groups, and IL-15 stimulation also did not affect IL-15Rα levels, suggesting that the differential regulation of IL-15 in Tregs might not be associated with its receptor expression. Second, the expression trends of IL-10 and IL-35, which are two important inhibitory cytokines produced by Tregs [48], were similar to Tregs, indicating the potential mediation of cytokine secretion in IL-15 regulation to Tregs. However, neither IL-10 nor IL-35 expression notably changed in CHB patients with T2DM in response to IL-15 stimulation. This is partly due to the synergistic effect of chronic viral infection and diabetes on immunological paralysis of T cells [49]. Third, although Hakim et al. found that IL-15 upregulated exhaustion markers PD-1 and TIM-3 on CD4^+^ and CD8^+^ T cells in healthy individuals [50], our current data suggest that IL-15 downregulated the expression of CTLA-4 and LAG-3 in Tregs in all groups. The controversy of the results might be due to the different disease status and different exhaustion markers. However, there was a different regulation of IL-15 to PD-1 levels in Tregs between controls and CHB patients with T2DM. This revealed that the difference of IL-15 regulation to Tregs might be due to PD-1 expression.

There are several limitations of the study. First, limited number of CHB patients with T2DM was enrolled in the study due to the low incidence. A large number of cases could be analyzed to obtain a suitable statistic for comparison. Second, there are three types of liver disease with diabetes [5]. However, it is hard to clinically diagnose to which category the CHB patients with T2DM belonged. Thus, we did not analyze the differences in cytokine expression and Tregs proportion among the three types. Third, circulating cytokines expression and peripheral immune cells might not accurately represent the status in the tissue microenvironments, such as in the liver and pancreas. Thus, further *in vivo* experiments or purified immune cells from liver or pancreas biopsy samples are needed to confirm the current results.

## Conclusion

T2DM and CHB might influence peripheral cytokine expression synergistically as well as independently. Both IL-7 and IL-15 were strongly suppressed in CHB patients with T2DM. Overall, insufficient IL-15 levels and decreased responsiveness of CD4^+^CD25^+^CD127^dim/−^ Tregs to IL-15 signaling might contribute to the immune dysfunction in CHB patients with T2DM.

**Conflicts of interest:** Authors declare no conflicts of interest.

**Funding:** Authors received no specific funding for this work.
